# Serotonin Syndrome in Parkinson’s Disease: Don’t Get It Confused by a Tremor

**DOI:** 10.7759/cureus.73963

**Published:** 2024-11-18

**Authors:** José Diogo Martins, Rita Morais Passos, Nuno Pardal, Ana Catarina Carvoeiro, Fernando Correia

**Affiliations:** 1 Internal Medicine, Unidade Local de Saúde do Alto Minho, Viana do Castelo, PRT; 2 Medical School, Universidade do Minho, Braga, PRT; 3 Critical Care Medicine, Unidade Local de Saúde do Alto Minho, Viana do Castelo, PRT; 4 Internal Medicine, Unidade Local de Saude do Alto Minho, Viana do Castelo, PRT; 5 Intermediate Care Unit, Unidade Local de Saúde do Alto Minho, Viana do Castelo, PRT

**Keywords:** geriatric pharmacology, intensive care unit (icu), monoamine oxidase inhibitors, serotonergic toxicity, serotonin syndrome (ss)

## Abstract

Serotonin syndrome (SS) is a potentially life-threatening condition caused by excessive serotonergic activity, often due to drug interactions. It classically manifests with autonomic and neuromuscular hyperactivity and by mental status changes that might include restlessness, delirium, and agitation. We present a case of a 76-year-old patient with Parkinson’s disease with SS triggered by interaction between rasagiline and buspirone. He presented with altered mental status, hyperreflexia, and muscle rigidity. Due to the severity of symptoms, he was early admitted to the ICU, and his clinical condition improved after discontinuation of serotonergic agents and supportive care. In SS, a high index of suspicion is fundamental, so early treatment is implemented.

## Introduction

Serotonin syndrome (SS) results from serotonin toxicity caused by excessive activation of serotonin receptors in the central nervous system (CNS), typically due to interference with the serotonin axis by exogenous drugs [1]. Common agents implicated in the syndrome include selective serotonin reuptake inhibitors (SSRIs), serotonin-norepinephrine reuptake inhibitors (SNRIs), and monoamine oxidase inhibitors (MAOIs) [1,2]. SS presents with a wide range of symptoms, from mild agitation to life-threatening hyperthermia, clonus, and autonomic hyperactivity [1-3]. Patients with Parkinson’s disease, frequently treated with MAOIs (like rasagiline), are at heightened risk when exposed to serotonergic agents because of the potential interaction between those medications [4].

In this report, we describe the diagnosis and management of SS in a Parkinson’s disease patient caused by the interaction of rasagiline and buspirone.

## Case presentation

A 76-year-old male with a previous medical history of Parkinson’s disease, alcoholic chronic liver disease, type 2 diabetes, essencial hypertension, and depression was regularly on the following therapy regimen: rasagiline (1 mg daily), buspirone (5 mg twice daily), levodopa-carbidopa, midodrine, lisinopril, amlodipine, and gliclazide. There had been no recent changes to his medication.

He was admitted to the emergency room due to altered mental status - psychomotor restlessness - generalized tremor and rigidity. According to relatives, he developed insomnia and confusion three days ago with worsening of symptoms and development of fever in the last 24 hours.

At physical examination, he had hyperthermia (T 39.0ºC), a blood pressure of 112/59 mmHg, heart rate of 118 bpm, and SpO_2_ of 99% in room air. Neurological examination revealed a Glasgow Coma Scale score of 10 (E1V4M5), generalized muscle rigidity, hyperreflexia, bilateral Babinsky sign, and roving eyes (Video 1). No focal neurological deficits were observed.

**Video 1:** Continuous eye movement ("roving eyes") in a patient with serotonin syndrome

A brain computed tomography (CT) scan was performed revealing no structural abnormalities. Due to the presence of hyperthermia and altered mental status, empirical antibiotics were started for possible meningoencephalitis. However, after lumbar puncture ruled out CNS infection that therapy was discontinued.

Given the mental status change, autonomic manifestations and neuromuscular hyperactivity associated with exposure to serotonergic medication, SS was strongly suspected. In this context, the patient was transferred to the general ICU for monitoring and supportive care.

At ICU admission, he maintained marked hyperreflexia and hyperthermia refractory to antipyretics (acetaminophen and diclofenac). In this context, sedation with midazolam was started, and given the high doses necessary, the medical team had to proceed to orotracheal intubation and mechanical ventilation.

During the first 24 hours, a liberal fluid resuscitation strategy was adopted to address rhabdomyolysis (Table 1).

**Table 1:** Laboratory workup of the patient at ICU admission

Parameters	Results	Reference values
Hemoglobine (g/dL)	8.3	11.8-15.8
Leucocytes (uL)	8840	4.0-10.0
Platelets (10^9^/uL)	110	150-400
Urea (mg/dL)	84	17-43
Creatinine (mg/dL)	1.39*	0.6-1.0
Creatinine kinase (UI/L)	3123*	30-200
Mioglobine (ng/mL)	5684*	1-147
Gamma-glutamyl transferase (UI/L)	70	<55
Aspartate transaminase (UI/L)	262*	8-35
Alanine transaminase (UI/L)	244*	10-45
C-reactive protein (CRP) (mg/dL)	1.64	0.01-0.82
International normalized ratio (INR)	1.31	<1.5

**Figure 1:**
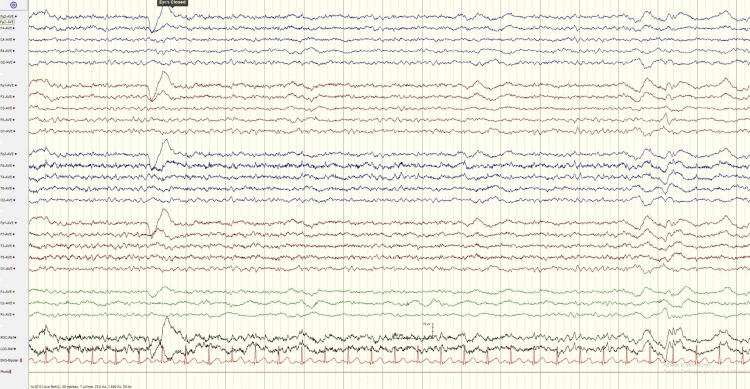
Electroencephalogram (EEG) with minor diffuse encephalopathy

**Figure 2:**
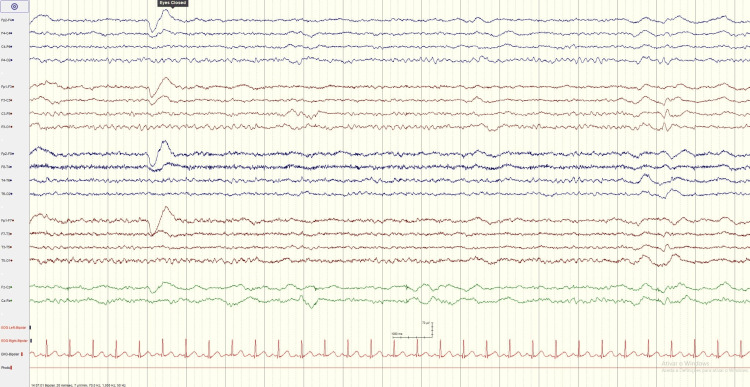
Electroencephalogram (EEG) with minor diffuse encephalopathy

At day 2, hyperthermia and neuromuscular hyperactivity started to improve allowing progressive weaning of sedation and daily spontaneous breathing trials since then. Midazolam was fully stopped at day 4. At day 7, an electroencephalogram was performed as the patient remained unresponsive. The exam had no evidence of seizure activity, showing minor encephalopathy (Figures 1-2). We integrated these findings into the recent infusion of midazolam in a patient with acute kidney injury. We kept a wait-and-see approach, and at day 9, he started to respond to simple commands. He was successfully extubated at day 12 to non-invasive ventilation, discontinued after 24 hours.

Upon transfer to the medical ward, the patient demonstrated progressive neurological recovery. His anti-Parkinsonian therapy was adjusted under the guidance of the neurology team, ensuring optimal symptom control without recurrence of serotonin syndrome. He was discharged with a therapeutic regimen that included levodopa/carbidopa for Parkinson’s disease management and quetiapine for sleep regulation, with a plan for further adjustments based on clinical and laboratory improvements.

The patient was transferred to a convalescent care unit to begin rehabilitation for disuse myopathy. At his internal medicine follow-up, he exhibited full functional recovery with no residual neurological or systemic sequelae.

## Discussion

SS is a rare but serious complication of serotonergic drug use, particularly in patients receiving MAOIs [1-3]. Our patient developed the syndrome due to the interaction between a MAOI and buspirone, a serotonin receptor agonist. The clinical presentation of hyperreflexia, muscle rigidity, tremor and hyperthermia align with the Hunter Serotonin Toxicity Criteria, although the presentation is not always that linear [5].

SS can mimic other conditions, such as neuroleptic malignant syndrome (NMS) and metabolic encephalopathy, making diagnosis challenging. However, the presence of clonus and hyperreflexia typically distinguishes SS from NMS, the last one characterized by lead-pipe rigidity. In Parkinson’s disease patients, the diagnosis is particularly challenging because of the overlapping symptoms of tremor and rigidity [5-6]. Early recognition of SS is essential, as the condition can lead to severe complications such as rhabdomyolysis, renal failure, and respiratory failure, all of which were present in this patient.

The management of SS focuses on discontinuing the causative drugs and providing supportive care, including sedation with benzodiazepines to control neuromuscular hyperactivity. In severe cases, mechanical ventilation may be required, as was the case. Fluid resuscitation is critical in cases of rhabdomyolysis to prevent renal failure [1-2].

## Conclusions

SS is a potentially life-threatening condition that requires prompt recognition and management, particularly in elderly patients with multiple comorbidities and complex medication regimens. Our clinical case highlights the importance of early recognition, discontinuation of serotonergic agents, and supportive care in preventing severe complications. Clinicians should exercise caution when prescribing serotonergic drugs, especially in combination with MAOIs, to avoid this serious condition.
